# Editorial: Emerging *Enterobacteriaceae* Infections: Antibiotic Resistance and Novel Treatment Options

**DOI:** 10.3389/fmicb.2017.00509

**Published:** 2017-03-28

**Authors:** Ghassan M. Matar

**Affiliations:** Department of Experimental Pathology, Immunology and Microbiology, Faculty of Medicine, American University of BeirutBeirut, Lebanon

**Keywords:** *Enterobacteriaceae*, resistance, ESBLs, carbapenemases, treatment

The high prevalence of diseases caused by organisms resistant to antibiotics in different regions of the world indicates an alarming global problem that requires rapid adequate action. Resistance to antimicrobials has been reported with a notable frequency in species belonging to the family *Enterobacteriaceae*, among other bacterial families, in sources including animals and food. For instance, the rate of fluoroquinolone-resistant *Salmonella* has rapidly increased in animals, food products, and human infections during the past few years. This has been correlated with the licensing of fluoroquinolone use in animal feed. This highlights the interconnectedness between various ecologies and supports the voices driving the global One Health initiative. In attempting to monitor current resistance trends in *Enterobacteriaceae*, the studies in the book at hand include surveillance information and patterns of transmission of resistant organisms to humans. This is in addition to providing possible guidelines that cater to particular agents, resistance mechanisms, and patient groups.

A study carried out over a period of 10 years by Hanna-Wakim et al. aimed to provide a comprehensive view of the epidemiological characteristics of urinary tract infections (UTIs) in hospitalized children, examine the risk factors of UTIs caused by extended spectrum beta-lactamase (ESBL)-producing organisms, and determine the resistance patterns in the isolated organisms.

The contribution by Moghnieh et al. focused on evaluating the epidemiology of bacteremia in cancer patients and emphasizing antibiotic resistance and risk factors of bacteremia associated with multi-drug-resistant organisms. They showed that emergence of resistance to third-generation cephalosporins and carbapenems in Gram-negative isolates has to be seriously considered in our local guidelines for empiric treatment of febrile neutropenia.

Liu et al. investigated the prevalence, resistance, and probable gene type of ESBL-producing *Escherichia coli* in China. The study further confirmed that ESBL producers, which are common among hospital strains of *E. coli*, are resistant to multiple drugs in addition to cephalosporins in China. This highlights the importance of closely monitoring such strains and providing scientific evidence for the rational application of antibiotics.

A study by Gao et al. evaluated the impact of ESBL-positive bacteria from animal manure on agricultural fields in rural regions of Taiwan, China. The study concluded that the application of animal manure carrying drug-resistant bacteria is a likely contributor to the spread of antibiotic resistance genes.

Zhang et al. attempted to understand the prevalence of CTX-M type ESBL-harboring *Enterobacteriaceae* and to analyze risk factors related to fecal carriage in healthy rural residents of Taiwan, China. The study concluded that the prevalence of fecal carriage of CTX-M ESBL-producing *Enterobacteriaceae* among healthy rural humans in Taiwan was high, and recent antibiotic use and hospitalization history may be important contributors.

A study done by Liao et al. focused on third-generation cephalosporins resistance in bacteria; they determined the characteristics and distribution of bla_CTX-*M-*14_, which encodes an ESBL, in *E. coli* harbored by food-producing animals in China. The study also suggested possible transmission of bla_CTX-*M-*14_ between animals and humans and proposed that the resistance gene context continues to evolve in *E. coli* of food-producing animals.

Animal to human dissemination of resistant bacteria, via the food chain or as a result of direct contact, has often been reported. Hence, Kilani et al. studied the occurrence of bla_CTX-*M-*1_, qnrB1, and virulence genes in avian ESBL-producing *E. coli* isolates from Tunisia. The authors also determined the antimicrobial resistance profile and the genomic variability of the isolates. They then concluded that plasmids harboring ESBL genes could be involved in the dissemination of certain resistance phenotypes.

Tokajian et al. also studied the molecular characteristics of an ESBL-producing *Klebsiella pneumoniae* strain, LAU-KP1, isolated from a stool sample from a patient admitted for a gastrointestinal procedure, by whole-genome sequencing. The entire plasmid content was also investigated. The study also questioned the potential role of *K. pneumoniae* as a reservoir for ESBL genes and other resistance determinants.

AmpC β-lactamases can hydrolyze broad- and extended-spectrum cephalosporins and are usually not inhibited by β-lactamase inhibitors such as clavulanic acid. Therefore, Hsieh et al. conducted a study that identified DHA-23, a novel plasmid-mediated and inducible AmpC β-lactamase obtained from *Enterobacteriaceae* isolates. They went on to characterize DHA-23 and compare it with other DHA molecules.

Insights into the resistance of carbapenems in two Gram-negative bacteria, *Raoultella ornithinolytica* and *Leclercia adecarboxylata*, are provided by Sun et al. They determined the nucleotide sequence of conjugative plasmids that encode the production of the enzyme New Delhi Metallo-beta-lactamase-1 (NDM-1), which is rarely isolated in these bacteria, from patients in China. The study also identified elements that facilitate transposition and mobilization of *bla*_NDM-1_ gene contexts.

Chen et al. studied carbapenem resistance in a specific carbapenem-non-susceptible *Enterobacter aerogenes* strain, 3-SP, isolated from a human case of pneumonia in a Chinese teaching hospital. The study aimed to determine the plasmid (pNDM-BJ0-like conjugative plasmid) that encodes for the NDM-1 carbapenemase production, termed p3SP-NDM. The plasmid was fully sequenced and compared with other similar pNDM-BJ0-like plasmids. All reported pNDM-BJ01-like plasmids are exclusively found in members of the genus *Acinetobacter*, whereas this is the first report of identification of a pNDM-BJ01-like plasmid in a member of the *Enterobacteriaceae*.

Malek et al. attempted to study the topic of emergence of resistance from a different perspective; they investigated the presence and distribution of class I and class II integrons and the characteristics of the gene cassettes they carry in *Enterobacteriaceae* from nosocomial infections at a University Hospital in Egypt. They determined their impact on resistance and identified risk factors for the existence of integrons. The study deduced that integrons carrying gene cassettes encoding antibiotic resistance are significantly present among *Enterobacteriaceae* isolates causing nosocomial infections.

Antimicrobials are usually avoided in the treatment of Shiga-toxin-producing *E. coli* (STEC) infections since they are believed to induce bacterial cell lysis and the release of stored toxins. The review by Rahal et al. summarizes the approaches to treatment of emerging STEC infections. The review presents alternative treatment approaches including the administration of toxin-directed antibodies, toxin-adsorbing polymers, probiotic agents, and natural remedies. The review also discusses how the use of antimicrobial agents in treating STEC infections has been reconsidered in recent years with certain modalities showing promise.

Small cationic peptides such as host defense peptides (HDPs) play a vital role in innate immunity response and immunomodulatory stimulation. In the study by Yacoub et al., the antimicrobial activities of β-defensin peptide-4 (sAvBD-4) and -10 (sAvBD-10) derived from chickens against pathogenic organisms including bacteria and fungi were investigated. The study then indicated that synthetic avian peptides exhibit strong antibacterial and antifungal activity. The authors concluded that future research should be carried out to better understand the mechanisms of action of these peptides and their potential use in the pharmaceutical industry.

In conclusion, it is rather evident that antibiotic resistance in members of the family *Enterobacteriaceae* is on the rise. The consistent stream of publications reporting resistant strains and sometimes novel resistance mechanisms has made it evident that infectious agents have become a threat to society yet again. The aforementioned reports are not constrained to a particular geographical location, and with the current ease of travel and global migration, the spread of resistant agents is a graver hazard than ever before. This is hence a worldwide problem that can only be curbed via diligent monitoring of arising resistance in various global regions, communication among the scientific community, formulating, and implementing appropriate guidelines, in addition to public education on the dangers of antibiotic misuse. With this in mind, the research topic at hand aimed at examining the current situation in geographical locations within the Far East, the Middle East, and North Africa. Lessons learned from this area of the world and observations in this global locale have often been different from those in the Western world, yet of mutual value. The articles encompassed by this topic also provide recommended guidelines for particular patient groups and infectious agents. Moreover, with a One Health mantra in mind, papers have included examination of soil and animal samples, hence stressing the relevance of arising resistance in such samples and its bearing on human health.

## Author contributions

The author confirms being the sole contributor of this work and approved it for publication.

### Conflict of interest statement

The author declares that the research was conducted in the absence of any commercial or financial relationships that could be construed as a potential conflict of interest.

